# ‘It’ll save your lungs’: early insights into nicotine pouch use and perceptions among young Australians

**DOI:** 10.1093/heapro/daae159

**Published:** 2024-11-21

**Authors:** Christina Watts, Bronwyn McGill, Shiho Rose, Amelia Yazidjoglou, Lorena Chapman, Anita Dessaix, Becky Freeman

**Affiliations:** The Daffodil Centre, Faculty of Medicine and Health, The University of Sydney, A joint venture with Cancer Council NSW, Newtown, New South Wales 2006, Australia; Charles Perkins Centre, Faculty of Medicine and Health, Prevention Research Collaboration, Sydney School of Public Health, The University of Sydney, Johns Hopkins Dr Camperdown, New South Wales 2050, Australia; Charles Perkins Centre, The University of Sydney, Johns Hopkins Dr Camperdown, New South Wales 2050, Australia; The Daffodil Centre, Faculty of Medicine and Health, The University of Sydney, A joint venture with Cancer Council NSW, Newtown, New South Wales 2006, Australia; Centre for Public Health Policy and Data, College of Health and Medicine, Australian National University, 62 Mills Road, Canberra 2601, Australian Capital Territory, Australia; Charles Perkins Centre, Faculty of Medicine and Health, Prevention Research Collaboration, Sydney School of Public Health, The University of Sydney, Johns Hopkins Dr Camperdown, New South Wales 2050, Australia; Cancer Council Western Australia, 1/420 Bagot Road, Subiaco 6008, Western Australia, Australia; Cancer Council New South Wales, 153 Dowling St, Woolloomooloo, New South Wales 1340, Australia; Charles Perkins Centre, Faculty of Medicine and Health, Prevention Research Collaboration, Sydney School of Public Health, The University of Sydney, Johns Hopkins Dr Camperdown, New South Wales 2050, Australia; Charles Perkins Centre, The University of Sydney, Johns Hopkins Dr Camperdown, New South Wales 2050, Australia

**Keywords:** vaping, e-cigarettes, tobacco, industry interference, commercial determinants of health

## Abstract

Flavoured oral nicotine pouches, manufactured and marketed by global tobacco companies, such as Philip Morris International (PMI) and British American Tobacco, entered the Australian market in 2024. Despite it being illegal for Australian retailers to sell nicotine pouches, the products have been seized in government enforcement raids of Australian retailers, and have also been promoted to young people by Australian social media influencers. The Australian Federal Government has recognized and expressed concern about the rising profile of nicotine pouches in Australia and the promotion of these products as unproven vaping and smoking cessation aids. Yet to date, there has been no published research on nicotine pouches in Australia. Using focus group and interview data collected in early 2024 as part of *Generation Vape*, an ongoing Australian national study into adolescent and young adult vaping and smoking, we explored their attitudes to and perceptions of nicotine pouches, including first-hand experiences and drivers of use. The participants perceived an increase in the use and popularity of oral nicotine pouches, including PMI’s Zyn brand, for reasons including claimed sport performance enhancement, vaping cessation and as part of partying and clubbing culture. Some participants perceived nicotine pouches to be a ‘healthier’ alternative to smoking or vaping. It is critical that research on nicotine pouches is collected now to inform public health policy and to prevent the proliferation of a new class of addictive recreational nicotine products in Australia.

**Contribution to Health Promotion**

- Nicotine dependence is a significant public health issue, increasingly among non-smoking young people.
- Our study provides the first insights into young Australians’ attitudes and perceptions of oral nicotine pouches, demonstrating that use may be growing for reasons such as sporting performance enhancement, as a vaping cessation aid, and as a party drug.
- Tobacco companies have a long history of adapting their products, or launching new product categories, in the face of growing regulations. The rise of nicotine pouches appears to be another example of this pattern.
- Research on nicotine pouch use is urgently needed to prevent further nicotine dependence.

## BACKGROUND

Globally, the use of flavoured oral nicotine pouches is growing (Majmundar *et al*., 2022; Tattan-Birch *et al*., 2022). Promoted as a ‘lower-risk’ and ‘tobacco-free’ nicotine product by manufacturers, including global tobacco companies Philip Morris International (PMI) and British American Tobacco (BAT), nicotine pouches deliver nicotine—either extracted from tobacco plants or formulated as a synthetic nicotine salt—via the mouth when placed between the lip and gum (Robichaud *et al*., 2020). From 2016 to 2020, nicotine pouch dollar sales increased by 305% in the USA (Marynak *et al*., 2021) and by 2026, the product market is expected to reach close to $33 billion (Gupta and Mehrotra, 2021). As of 2024, nicotine pouches are legally sold in at least 33 countries of which 13 have no specific regulations at all (Duren *et al*., 2024).

The growth in nicotine pouch sales globally has occurred alongside concerted marketing efforts, positioning the products as safer than combustible tobacco products, and easier to use. In 2019, the top six nicotine pouch brand manufacturers spent $11.2 million on advertising campaigns in the USA (Ling *et al*., 2023). A 2021 US study found that nicotine pouches were the second most prevalent nicotine product used by adolescents (with vaping products most common) (Harlow *et al*., 2022). A UK study found that while nicotine pouch use is currently relatively low, the products have become more popular over time, with use concentrated among younger people (Tattan-Birch *et al*., 2022). This targeting is yet another example of the disproportionate impact of the commercial determinants of health on young people. Tobacco use and nicotine addiction amongst young people must be considered within the broader context of harmful industry practices that evade and manipulate public health policies.

There are emerging and potential health hazards associated with nicotine pouches. Chemical testing in Germany showed that more than half of a sample of nicotine pouches contained carcinogenic tobacco-specific nitrosamines (Mallock *et al*., 2024). The products also contained more than 50 mg of nicotine per pouch—a concentration that is likely to induce and maintain nicotine dependence (Mallock *et al*., 2024). Prolonged and regular use of nicotine pouches is also expected to increase the risk of local oral health problems (Jackson *et al*., 2023). Since nicotine pouches can be discreetly used, have high nicotine concentrations and are sold in a variety of appealing flavours, such as citrus, cherry, peppermint and coffee (Robichaud *et al*., 2020), there is a risk of rapid uptake by non-smoking young people and ongoing nicotine dependence in the population.

Nicotine pouches appear to have entered the Australian retail market in late 2023, despite being illegal to sell. Unlike nicotine replacement therapies such as patches, lozenges and gum, nicotine pouches have not been evaluated for quality, safety and efficacy in Australia and therefore are not included in the Australian Register of Therapeutic Goods, meaning it is illegal to market or sell these products in Australia (Department of Health and Aged Care, 2024). There is no data to suggest that nicotine pouches serve as an effective cessation aid for either vaping or smoking (Australian Department of Health and Aged Care, 2024). The Australian Federal Government has recognized the rising profile of nicotine pouches in Australia and the illegal promotion of these products as smoking cessation aids (Department of Health and Aged Care, 2024; May and Butler, 2024). Since January 2024, 1.3 million nicotine pouches have been seized at the Australian border, which is 950% more than the total number of nicotine pouches seized in the 2 years prior (Perpitch, 2024). Nicotine pouches have also been seized in government enforcement raids of Australian retailers (Theocharous, 2024), and have been promoted to young people by Australian influencers on TikTok and Instagram (May, 2024).

Despite news media reports of young people using nicotine pouches and being the target of industry marketing activity (May, 2024; May and Butler, 2024), there is limited research about nicotine pouch use in Australia. A recent Australian study found that 19% of a sample of 1598 Australians aged 16–39 years had used nicotine pouches in the past 30 days (Jongenelis *et al*., 2024). It is essential that adolescent and young adult behaviours, attitudes and perceptions relating to nicotine pouch use are closely monitored to both detect and prevent use. Youth vaping in Australia grew rapidly to become a national public health crisis, with lifetime use of e-cigarettes among 14- to 17-year-olds increasing from 9.2% in 2016 to 28.4% in 2022–23. Similarly, 21% of 18- to 24-year-olds vaped daily in 2023, up from 5% in 2019 (Australian Institute of Health and Welfare, 2024). There is a significant risk that nicotine pouch use could become a similar problem, particularly if young vape users switch to these products following the October 2024 national Australian reforms that allow vape sales to adults as behind-the-counter medicines in pharmacies only. People under age 18 will require a prescription to access a vape, a measure designed to reduce the accessibility of the products to young people (Therapeutic Goods Administration, 2024b).

There is no Australian research on nicotine pouches and evidence is urgently needed to inform regulatory and public health responses This study explores young adults’ and adolescents’ attitudes and perceptions of nicotine pouches, including first-hand experiences and reasons for use.

## METHODS

This study uses data collected as part of *Generation Vape*, an ongoing Australian national study into adolescent and young adult vaping and smoking. The Generation Vape study commenced in 2021 and involves 6-monthly cross-sectional surveys of Australian adolescents aged 14–17 years, parents and guardians of 14- to 17-year-olds, secondary school teachers, and young adults aged 18–24 years, as well as yearly qualitative research (interviews and focus groups) with each of these cohorts. Several papers from the Generation Vape study have been published elsewhere (Watts *et al*., 2022; Egger *et al*., 2024) and in *Health Promotion International* (Yazidjoglou *et al*., 2024). The current study reports on qualitative data from Wave 6 (February–April 2024) with adolescents and young adults who vaped or had tried vaping in the past. In this wave of Generation Vape, the topic of nicotine pouches was often raised spontaneously/unprompted and highlighted as a key area of exploration. As such, this study analysed perceptions and experiences of nicotine pouches.

### Recruitment

Recruitment of adolescent and young adult participants was conducted by a professional research recruitment agency following procedures approved by the University of Sydney Human Research Ethics Committee. Adolescents and their parent/guardian, and young adults, were all provided with Participant Information Statements and consent forms prior to participation. For adolescents, permission to participate in the study was granted by their parent or guardian. As a reimbursement for their time, adolescents received a $45 gift card, and young adults received a $100 gift card. These reimbursements could not be used on tobacco or alcohol. Participant segmentation was applied to ensure heterogeneity of sex (male/female), location (state and metro/regional areas), school type for adolescents (private/government schools), smoking status for young adults (smoker/non-smoker) and vaping status (trial, former, occasional and regular vaper) for both adolescents and young adults. ‘Trial’ vaper included those who had vaped previously, less than 10 times in total, but not in the past month. ‘Former’ vapers were those who have vaped previously, at least 10 times in total, but not in the past 3 months. ‘Occasional’ vapers had vaped at least 10 times in total, and within the past 3 months, and ‘Regular’ vapers were those who had vaped at least 10 times in total, within the past month, and at least once per week. In this wave of data collection, we intentionally only recruited current, former and trial-only vapers to gain a deeper understanding of the perceptions, attitudes and behaviours of those with experience vaping.

### Data collection

Semi-structured interviews were conducted with 98 adolescents either one-on-one or in pairs (78 interviews total). A third-party agency with extensive experience in qualitative research in public health facilitated 22 semi-structured focus groups with 112 young adults using Zoom video conferencing. Interviews and focus groups lasted approximately 45 and 90 min, respectively. All interviews and focus groups were audio-recorded and transcribed verbatim with participants’ consent. Discussion guides for the interviews and focus groups were developed and piloted by the research team. The guides were designed to explore knowledge, attitudes and perceptions of vaping products, their health impacts and policies and regulations, along with product use behaviours. Young adults were also asked about their knowledge, attitudes, perceptions and behaviours related to tobacco smoking. While no questions were directly asked about nicotine pouches in the discussion guides, three adolescents and 19 young adults raised nicotine pouches, unprompted, when asked about vape laws, products and promotions, and vaping quit attempts. The facilitator probed for further information and clarity when the topic was raised.

### Analysis

We used the Framework Method to analyse the data (Gale *et al*., 2013) as it is a transparent and systematic process and suitable for applied research (Goldsmith, 2021). The themes and concepts were developed inductively from participant discussions of nicotine pouches. The research team familiarized themselves with the data by reading and re-reading the transcripts (C.W., B.F.), as well as listening to the audio recordings (C.W.). C.W. initially coded the transcripts using NVivo 14 and discussed the codes with B.F. to determine a final analytical framework to code all the data related to nicotine pouches within the transcripts.

### Ethics

Ethics approval was provided by the University of Sydney Human Research Ethics Committee (Project number: 2021/442).

## RESULTS

### Growing use and popularity among young people

Discussions around nicotine pouches and their use and popularity among young people were highly varied. While some participants were not aware of nicotine pouches at all, others noted they were aware of nicotine pouches and their use but had not personally tried them. Several participants highlighted that use was growing among their peers, and in some cases, overtaking vaping in popularity, suggesting that social norms around use could be rapidly changing:

*I would say that’s like at least in my circle, almost starting to overtake vaping, because everyone’s tryna [sic] now quit vaping.* (24-year-old, Female, trial vaper, had not used nicotine pouches)

*Well recently they’ve kind of gone big, like, even people that don’t vape have gone “oh yeah try out these Zyn* [PMI’s nicotine pouch brand]*”. Everyone’s making like a massive deal about em*. (17-year-old, Male, regular vaper, had tried nicotine pouches)

The Australian vaping reforms introduced in 2024 banned the importation of vaping products and disposable devices, and limited the supply of therapeutic vapes to pharmacies only with strict controls on packaging, flavours and nicotine concentrations. Several participants were of the belief that the growing use of pouches was a direct consequence of these vaping reforms, or that the new laws would drive up pouch use even further. Participants also expressed the notion of novelty and newness being an important factor in the face of vapes being more difficult to access:

*…my other thought would be yeah, a lot of people are gonna move to like ‘Zyn’s’, which are the like nicotine pouches that you can stick up in your gums, I think they’re already trending, and that’s gonna go up.* (24-year-old, Male, occasional vaper, had tried nicotine pouches)

*Yeah, I think, definitely like the crackdown on it has been [sic] an effect, like I don’t know if anyone else has seen, but these like little nicotine pouches that you stick in your gums um definitely in my area are becoming way more popular, like vapers are sorta [sic] switching to them now, and in like the exact same trend as in when vapes came, um everyone wants them now. Um, and also I think just the novelty of it [vaping] has worn off, like I just sort of, it’s not this new thing that wasn’t around before, it’s just been around for a while, and I think people sort of a bit, yeah the novelty’s worn off.* (23-year-old, Male, occasional vaper, nicotine pouch use status unknown

### Drivers of use

Several participants offered views on why nicotine pouches were used by young people, and the situations in which they are used. There was a wide range of drivers of use suggested by young people. For example, nicotine pouches were discussed as being used during sport for performance enhancement by some participants:

*Yeah, they use them a lot in elite sports, cause like obviously the nicotine like kind of wakes you up a bit, they use them for that like alertness, and with yeah, so we’ve used them, I’ve used them like in a game of soccer before.* (21-year-old, Male, regular vaper, had tried nicotine pouches)

*Like I know some, like there’s a lot of athletes that like take ‘em, as like a nicotine pouch, just like get them through a game and stuff like that.* (21-year-old, Male, trial vaper, had not tried nicotine pouches)

*I heard about in a sporting context, ah can’t recall much of it, but probably ah yeah thinking that was a bizarre way, and how, ah, I guess drug testing and that sorta thing, if it had any play into that.* (22-year-old, Male, trial vaper, had not tried nicotine pouches)

In contrast to the highly specific driver of use for sport performance, others explained that nicotine pouches were being used on nights out ‘partying’—at clubs and festivals, for example:

*…people have started using those on nights out, because they’ve been able to get them in Australia somehow, and I’ve seen that at festivals… You pretty much like stick ‘em up in your gum. And then, you’re set for the night apparently.* (21-year-old, Male, trial vaper, had not tried nicotine pouches)

*I haven’t tried them but I know of them, yeah. I know they’re big on night outs and stuff.* (18-year-old, Male, regular vaper, had not tried nicotine pouches)

For one participant, nicotine pouches were described as akin to a party drug:

*Oh yeah I got [inaudible] off some guy in a club once, it just made me go nuts. [Interviewer: So, would you do it again?] Um, if I wanted to get a bit loose, yeah I would.* (24-year-old, Male, regular vaper, had tried nicotine pouches)

Other discussions around the reason for using nicotine pouches reflected the growing concerns of vaping addiction and the desire to quit amongst young people (Freeman *et al*., 2024) with some participants suggesting they were a possible vaping cessation tool. It was noted by multiple participants that pouches were being used to help people quit vaping, often to control nicotine cravings, or as an alternative nicotine product for use:

*[Interviewer: The time when you were stopped (vaping) for a couple of months, how did you cope?] Um well, the time that I lasted three months, I used these other things. Like they’re nicotine pouches and you put in em in your gum. And that like kinda got away from the urge of vaping but like I still wanted to do it, but I don’t know I just every time I wanted to, I’d just have one of them and then it’d get rid of that want… So, I was like oh yeah might give it a go cos it’s got nicotine, and it might stop me from wanting a vape.* (17-year-old, Male, regular vaper, had tried nicotine pouches)

*[Interviewer: Is there anything else you can think of that would help you, or assist you in quitting if you wanted to?] I wanna try Zyns. That’s about it.* (14-year-old, Male, regular vaper, had not tried nicotine pouches)

*[Interviewer: You said if you could magically stop (vaping) you just would. Could you imagine what would be able to help you?] Zyns. [Interviewer: Other than Zyns?] A lot of Zyns.* (14-year-old, Male, regular vaper, had tried nicotine pouches)

*A lot of my friends now are like kind of wanting to stop (vaping), um one thing I have noticed is I don’t know what these are called, but they’re kinda now swapping to these things that you like put in your gum, ah I don’t know what they are, but um, I know like all of my friends’ kind of boyfriends are more so than the girls, are really into these like, I think they’re from Sweden or something.* (24-year-old, Female, trial vaper, had not tried nicotine pouches)

While some participants described using nicotine pouches to help them quit vaping, there were no personal accounts from former vapers who had used the products to successfully quit smoking or vaping.

### Health perceptions

Some participants who had personally used nicotine pouches offered insights into their own experiences using the products. A range of both negative and positive experiences were described with pouch use. Of those who had a negative reaction to pouches, this included feeling sick, gums burning, and not enjoying the taste:

*My mate brought a packet to the gym two days ago, and they were full-strength ones and I put two in my mouth, and then yeah, I had that, I got nic-sick. But like it wasn’t, I just took them out but I put them in, 5 minutes later it was just like, I just felt sick straight away.* (18-year-old, Male, regular vaper, had tried nicotine pouches)

*[Interviewer: What’s good about them?] Nothing, I hate em… They’re just like, ‘cause they burn your gums and they just make you feel a little bit sick sometimes and they taste like shit pretty much.* (17-year-old, Male, regular vaper, had tried nicotine pouches)

Given the focus of the research on vape use, some participants who had tried nicotine pouches, also made comparisons to vaping products. Pouches were not considered as desirable or enjoyable as vapes:

*It’s alright, but it doesn’t really have the same effect (as vaping), it sort of just made me feel shit-house. And then um, and yeah, I guess it wouldn’t really work in replacing it ‘cos you’re sort of just giving yourself the same thing that’s keeping you addicted*. (19-year-old, Male, regular vaper, had tried nicotine pouches)

*I tried the new product, it’s called a ‘Zyn’ or something like that, and it’s a nicotine pouch instead, where it goes under your gum. [Interviewer: How did that go?] Doesn’t really do much, so it’s like, it, the sensation of vaping is like stronger, and I don’t know. [Interviewer: Did it meet your needs?] It, nah it didn’t, like the vaping felt better than the alternative products…. I vaped when it was in my mouth. So like, I felt like I was doing worse for myself, than before.* (21-year-old, Male, regular vaper, had tried nicotine pouches)

The positive experiences with pouch use emphasized the perceived benefits of pouch use over vapes or cigarettes. Some of the participants described pouches as a way of getting nicotine ‘hit’ without impacting lung health:

*Honestly yeah, it’s a healthier solution. It’ll save your lungs…* (14-year-old, Male, regular vaper, had tried nicotine pouches)

*A quick nicotine hit, without the smoking part*. (21-year-old, Male, Regular vaper)

*Participant A: If there was like a healthier, like, version of it [vapes]. Participant B: Have you tried Zyns? Participant A: Ah yes, Zyns, I didn’t even think about that.* (Dialogue between two 14-year-old, Males, Regular vapers, Participant A had tried nicotine pouches, Participant B had not tried them)

## DISCUSSION

Our study provides the first evidence of young Australians’ perceptions of and reasons for using flavoured oral nicotine pouches. It appears that the product is used for sport performance enhancement, on nights out clubbing and partying, and in some cases, to assist with nicotine cravings and vaping cessation. While seen in a negative light due to the adverse effects of use by some, others believed that nicotine pouches provided a healthier alternative to vaping and/or smoking. This finding aligns with US research that found nicotine pouches were perceived as less harmful and less addictive than cigarettes, e-cigarettes and smokeless tobacco products (Tosakoon *et al*., 2023). Such favourable beliefs about nicotine pouches have been reported to predict susceptibility to and current use of the products (Sparrock *et al*., 2023).

Positive health perceptions related to nicotine pouches may originate from the tobacco industry’s promotion of these products within their ‘harm reduction’ product portfolios. Studies examining the marketing messages from leading nicotine pouch brands, including PMI’s Zyn brand, showed that advertising headlines often feature health claims such as ‘tobacco-free’ or ‘smoke-free’ (Duan *et al*., 2024), which have the potential to falsely alleviate consumers’ concerns about the potential health harms associated with use (Ling *et al*., 2023). While some participants were of the belief that nicotine pouches are ‘safer’ or ‘healthier’ than perhaps tobacco or vaping, and that they could be used to aid vaping or smoking cessation, there is limited to no evidence of the health harms associated with these products, nor on their effectiveness as cessation aids.

The emergence of nicotine pouches on the Australian market coincides with significant regulatory reforms to control access to vaping products in response to growing concerns about the rapid rise in adolescents and young adult vaping product use (Therapeutic Goods Administration, 2024b). To curb use by non-smokers and younger Australians, a ban on all disposable vaping products was implemented in January 2024. Further to this, on 1st July 2024, a ban on the commercial retail sale of all vaping products outside of pharmacies commenced. During a transition period, between July and September 2024, everyone required a prescription to access vapes. Then, as of 01 October 2024, adults are able to access vapes without a prescription from pharmacies (Therapeutic Goods Administration, 2024a). Some Australian states, Western Australia and Tasmania, for example, have indicated that they intend to retain/reestablish the prescription-only access approach to vapes and will not be allow behind-the-counter sales. Products supplied through pharmacies have minimum quality standards, such as maximum nicotine concentrations, limited flavours and standardized medical-style plain packaging. The pharmacy-only model of access to vaping products in Australia is a significant threat to the tobacco industry. Tobacco companies like PMI and BAT, that both have nicotine pouches on the market, rely on nicotine addiction in younger generations to support their future revenue-raising prospects as tobacco smoking declines. The emergence of flavoured oral nicotine pouches, and youth-targeted promotions in Australia at a similar time to the sales restrictions on vapes is unsurprising. Tobacco companies have a long history of adapting their products, or launching new product categories, in the face of growing regulations, and the rise of nicotine pouches in Australia is yet another example of this pattern.

While our study provides the first insights into nicotine pouch use in Australia, it is critical that further research is conducted to comprehensively understand how the products are used, promoted and sold, their potential toxicity, and to track the prevalence of use among all population groups. Addiction to new nicotine products, such as nicotine pouches, is a significant public health issue, particularly among young people. Proactively researching this topic, and rapidly delivering evidence to policymakers, will enable policymakers to be on the front foot to respond to the issue with the necessary speed needed to prevent further nicotine addiction in the population. Historically, research on new nicotine-containing products marketed and sold by transnational tobacco companies, like vaping products, has occurred well after the products first appeared on the market. For example, in the case of vaping product use in Australia, it has taken several years for research to understand the extent of the problem and what laws and regulations needed to be implemented and tightened, lagging well behind industry product and marketing innovations. Although nicotine pouches cannot be legally sold in Australia at present, the products still appear to be reaching the hands of young people, indicating that the current policy and enforcement approach needs to be monitored and reviewed to ensure it is effective in preventing the uptake of these products.

### Limitations

There is a possibility of recruitment bias given the recruitment of participants from online panels, however, this study is exploratory in nature and provides opportunistic insights that can help guide future research. Given the topic of nicotine pouches was raised by participants without prompts from the discussion guide (i.e. the primary purpose of the study was to explore attitudes, perceptions, knowledge, attitudes and behaviours related to vapes and tobacco, and not nicotine pouches), significant gaps in knowledge remain. Future waves of Generation Vape will more thoroughly explore the topic of nicotine pouches with adolescents and young adults.

## CONCLUSION

Young Australians perceive an increase in the use and popularity of oral nicotine pouches, including PMI’s Zyn brand, for reasons including sport performance enhancement, vaping cessation and as part of partying and clubbing culture. Given the recent rapid increases in vaping prevalence among adolescents and young adults in Australia, and major regulatory reforms to curb these rates, the entry of nicotine pouches on the Australian market is a public health concern requiring urgent research and further regulatory attention. There is a unique and timely opportunity to gather evidence on nicotine pouches to inform policy approaches now, prior to a potential spike in use, as seen with vaping amongst young Australians in recent years.

## DATA SHARING

The data underlying this article cannot be shared publicly to protect the privacy of individuals who participated in the study.
